# 

**Published:** 1857-07

**Authors:** 


### CONTENTS.

ORIGINAL COMMUNICATIONS— PAGE.

Report of the Committee on Practical Medicine. By F. R. Payne, ...... 295

Cook County Medical Society, ...... 315

Case of Extensive Injury by Machinery, to the Upper Extremity. By Thos. Hall, M. D., ...... 322

BOOK NOTICES—

An Explanation of the Signs and Symptoms of Pregnancy, with some other Papers Connected with Midwifery. By W. F. Montgomery, A. M., M. D., etc.,...... 323

PROCEEDINGS OF MEDICAL SOCIETIES—

American Medical Association, at Nashville, ...... 332

Illinois State Medical Society, ...... 336

Fayette County Medical Society, ...... 337

EDITORIAL—

To Subscribers, ...... 338

Rush Medical College, ...... 338

Advertisement, ...... 339

MISCELLANEOUS ITEMS—

Death of Dr. Thomas Spencer, ...... 340

Medical Schools—Medical Politics, ...... 340

Editorial Changes, ...... 341

